# Compositional analysis of the association between 24 h movement behaviours, HbA_1c_ and interstitial glucose in children and adolescents with type 1 diabetes mellitus: a two-year longitudinal analysis of the Diactive-1 cohort study

**DOI:** 10.1007/s00125-025-06496-2

**Published:** 2025-07-31

**Authors:** Jacinto Muñoz-Pardeza, José Francisco López-Gil, Ignacio Hormazábal-Aguayo, Nidia Huerta-Uribe, Yasmin Ezzatvar, Antonio García-Hermoso

**Affiliations:** 1 Navarrabiomed, University Hospital of Navarra, Public University of Navarra (UPNA), IdiSNA, Pamplona, Spain; 2 School of Medicine, Universidad Espíritu Santo, Samborondón, Ecuador; 3 Department of Communication and Education, Universidad Loyola Andalucía, Sevilla, Spain; 4 Vicerrectoría de Investigación y Postgrado, Universidad de La Serena, La Serena, Chile; 5 Lifestyle Factors with Impact on Ageing and Overall Health (LAH) Research Group, Department of Nursing, University of Valencia, Valencia, Spain; 6 Vicerrectoría de Investigación y Postgrado, Universidad de Los Lagos, Osorno, Chile

**Keywords:** HbA_1c_, Insulin-dependent diabetes, Physical activity, Reallocating analysis, Sedentary behaviour, Sleep duration

## Abstract

**Aims/hypothesis:**

The aim of this study was to examine the association of physical activity, sedentarism and sleep with HbA_1c_ and interstitial glucose in children and adolescents with type 1 diabetes through a 24 h compositional analysis.

**Methods:**

The study involved 83 young people diagnosed with type 1 diabetes (aged 6–18 years; 45% girls, mean HbA_1c_ 57.54 ± 9.22 mmol/mol (7.4 ± 0.8%); median interstitial glucose 9.37 mmol/l [IQR 8.68–10.31]) from the Diactive-1 cohort study, followed up for 2 years. A triaxial accelerometer was used to objectively measure 24 h movement behaviours for 9 days. HbA_1c_ levels were obtained from medical records, and interstitial glucose data were collected through continuous glucose monitoring. Linear mixed models were used to quantify associations between movement behaviours, interstitial glucose and HbA_1c_, maintaining the relative nature of the data based on the 24 h day.

**Results:**

A higher daily amount of sedentary behaviour, at the expense of sleep time, light or moderate-to-vigorous physical activity, was positively associated with HbA_1c_ (unstandardised beta coefficient [*B*]=14.077 [95% CI 4.244, 23.956]; standardised beta coefficient [*β*]=0.368) and interstitial glucose (*B*=1.988; 95% CI 0.153, 3.880; *β*=0.261), while more sleep time, at the expense of sedentary behaviour, light or moderate-to-vigorous physical activity, was associated with a significant reduction in HbA_1c_ (*B*=−12.712; 95% CI −25.204, −0.520; *β*=−0.197). Furthermore, reductions in both interstitial glucose (*B*=−1.580; 95% CI −2.800, −0.388; *β*=−0.283) and HbA_1c_ (*B*=−9.361; 95% CI −15.856, −2.881; *β*=−0.330) were observed with increased daily time spent in moderate-to-vigorous physical activity at the expense of sedentary behaviour. Overall, the standardised beta coefficients indicated small to moderate effect sizes.

**Conclusions/interpretation:**

Our findings indicate that lower sedentary behaviour and more optimal sleep and physical activity patterns are associated with greater metabolic stability in children and adolescents with type 1 diabetes. These findings support the need for further research on balancing these behaviours for better diabetes management, and encourage adoption of a 24 h movement approach in clinical care.

**Graphical Abstract:**

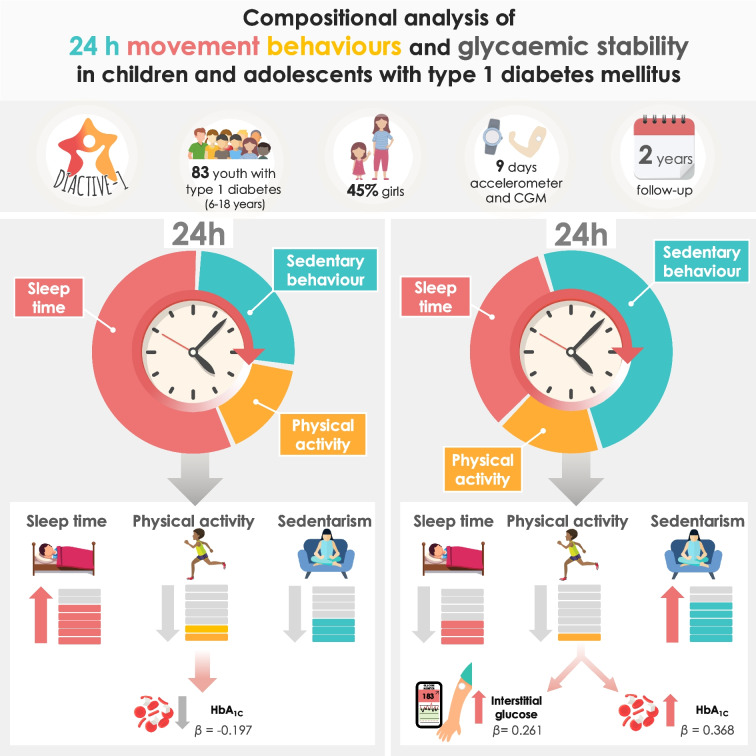

**Supplementary Information:**

The online version contains peer-reviewed but unedited supplementary material available at 10.1007/s00125-025-06496-2.


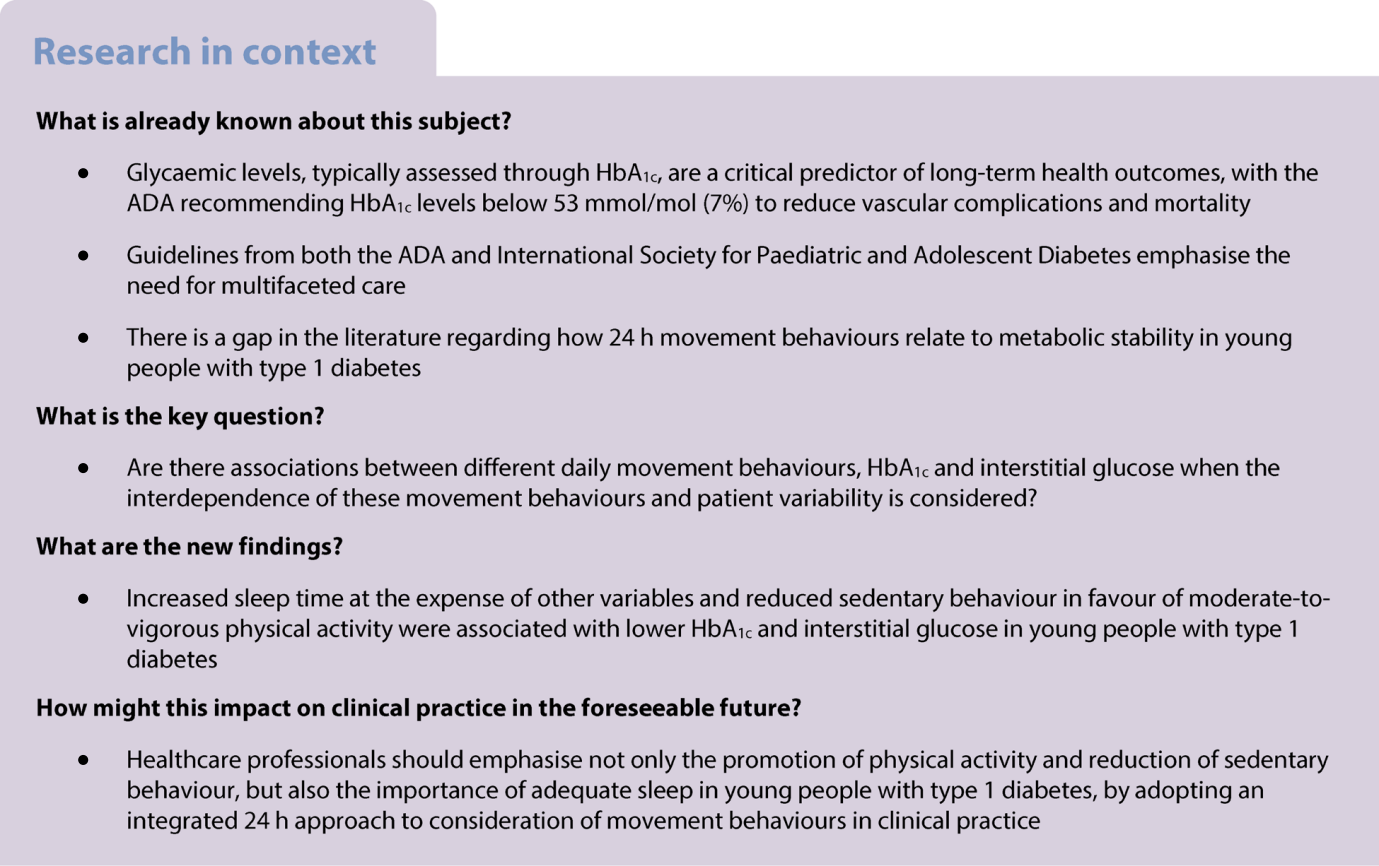


## Introduction

Type 1 diabetes mellitus is an autoimmune and inflammatory disease that was initially considered to affect only children and adolescents; however, it is now widely recognised that it can be triggered at any age [1]. This condition affects approximately 1.4 million children and adolescents [2], with a global incidence rate of 14.07 per 100,000 person-years [3]. It is characterised by progressive destruction of pancreatic beta cells, leading to insulin deficiency [4], and resulting in glycaemic impairments that increase the risk of microvascular and macrovascular complications [5]. Management of glucose levels [6], often assessed through HbA_1c_ or blood/interstitial glucose, is an important predictor of long-term health outcomes due to its strong associations with these complications [7]. In line with this, the ADA guidelines recommend maintaining HbA_1c_ levels below 7% to reduce vascular complications and mortality [8, 9]. Similarly, ADA has also reported that achieving glycaemic targets, including maintaining stable glucose levels, can mitigate the risk of acute and chronic complications [10], and has also been associated with future HbA_1c_ levels [11].

ADA and the International Society for Paediatric and Adolescent Diabetes recommend multifaceted care for paediatric patients with type 1 diabetes, including physical activity and exercise [12]. They advise at least 60 min of moderate-to-vigorous physical activity (MVPA) per day while minimising sedentary behaviour, guidelines that align with those established by the WHO [13]. Young people who adhere to these guidelines can reduce their HbA_1c_ level, thereby improving metabolic stability [14].

Huerta-Uribe et al [15] reported that physical activity was associated with lower HbA_1c_ levels, whereas sedentary time was positively associated with HbA_1c_ levels. Studies with large sample size have also demonstrated reductions in glucose levels with regular physical activity [16], with MVPA emerging as a predictor of glycaemic stability over sustained periods of 2 weeks [17]. Additionally, long-term engagement in physical activity has been linked to improved glycaemic profiles [18] and better future HbA_1c_ outcomes in this population [19]. There is also evidence highlighting the role of sleep in glycaemic stability. Cross-sectional studies have found that children and adolescents with type 1 diabetes who had higher HbA_1c_ levels experienced shorter sleep duration [20] and sleep irregularities [21]. Evidence also suggests that individuals with type 1 diabetes and elevated HbA_1c_ and glucose levels experience shorter sleep duration compared to their apparently healthy peers without diabetes [22]. However, these studies assume a linear and infinite relationship between time spent on one behaviour and diabetes-related parameters, without considering the possible compositional interplay between sleep, physical activity and sedentary behaviour. Furthermore, they overlook the finite nature of the day, whereby time allocated to one activity inevitably reduces the time available for others [23].

A systematic review by Patience et al [24] emphasised the importance of a comprehensive 24 h approach to managing type 1 diabetes. This review examined each component of the 24 h movement behaviour model individually, including physical activity, sedentary behaviour and sleep, while also highlighting the absence of studies assessing these behaviours collectively for their combined impact on metabolic stability. This lack of studies underlines the need for research that considers the synergistic effects of movement behaviours throughout the day on type 1 diabetes outcomes [24]. Therefore, the aim of this study was to examine the association of physical activity, sedentary behaviour and sleep duration with HbA_1c_ and interstitial glucose in children and adolescents with type 1 diabetes through a 24 h compositional analysis. The study hypothesis was that reallocation of time from sedentary behaviour to physical activity, as well as increased sleep duration, would be associated with reductions in HbA_1c_ and lower interstitial glucose.

## Methods

### Study design

The Diactive-1 cohort study is an ongoing longitudinal study evaluating children and adolescents diagnosed with type 1 diabetes and living in the autonomous community of Navarra (Spain) across three assessment time points over 2 years. This study was approved by the Research Ethics Committee on Medicines of the University Hospital of Navarra (PI_2021/32) and was conducted in accordance with the principles outlined in the Declaration of Helsinki (2013).

### Setting

Recruitment for the study was conducted at the University Hospital of Navarra from May 2021 to February 2022. Before starting the evaluations, the legal guardians gave their written consent, and the children and adolescents signed an assent form to indicate their voluntary agreement before the baseline measurements. At each subsequent evaluation, they were contacted by telephone to schedule a new appointment, at which time they could choose to continue or withdraw voluntarily without giving a reason. The baseline and both follow-up assessments were conducted annually over a two-year period in a specialised youth-friendly physical exercise laboratory at the Navarrabiomed Biomedical Research Centre and supervised by a multidisciplinary team. The measuring instruments were operated solely by specialised technicians who were unaware of any information related to the participants. The duration of each evaluation was approximately 90 min each, and they were scheduled for early in the morning to ensure precise monitoring of glucose levels.

### Participants

**Fig. 1:**
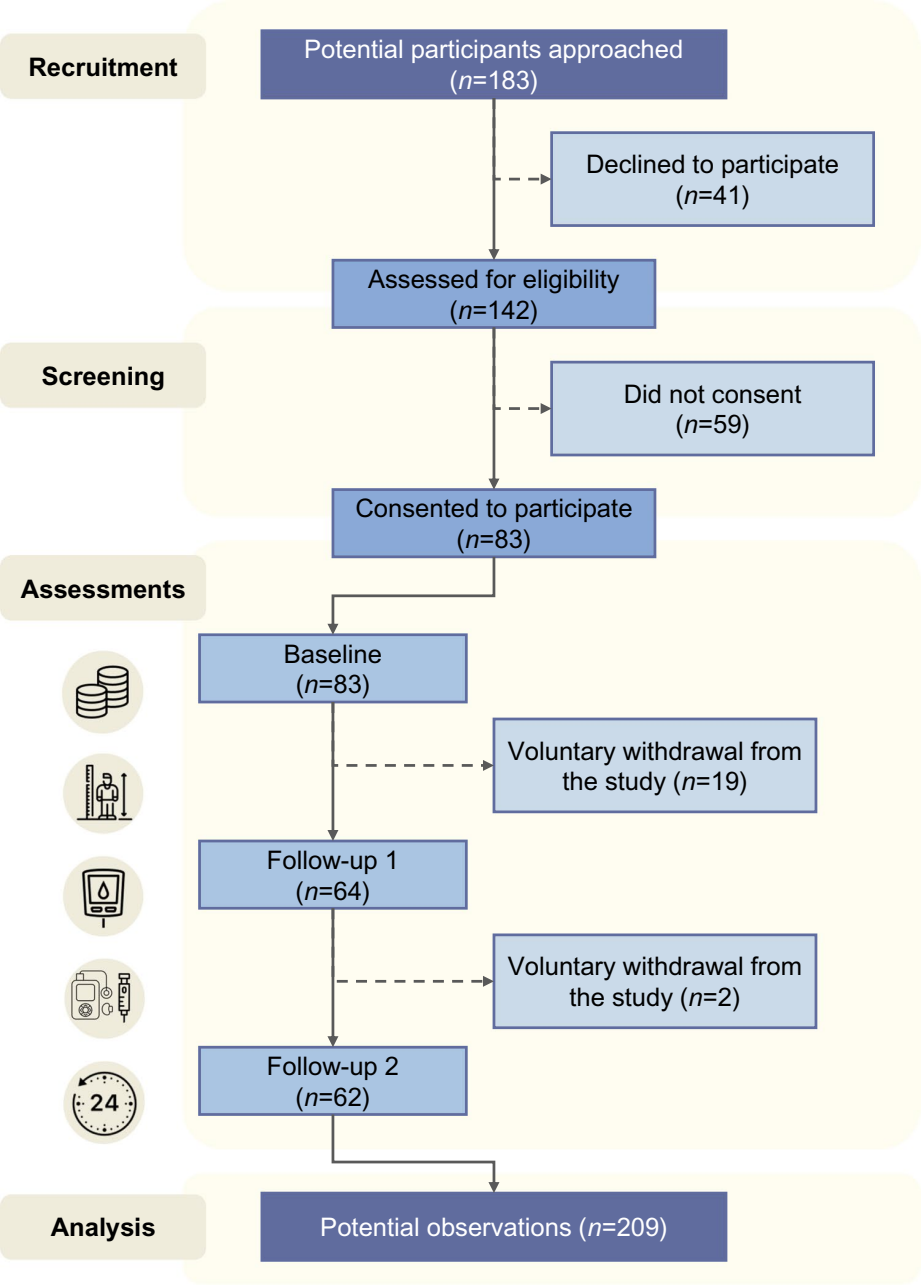
Study flowchart showing recruitment, screening and assessments, leading to 209 potential observations across the baseline and two follow-up time points

Young people aged 6–18 years who had been diagnosed with type 1 diabetes for more than 6 months participated. Children were defined as individuals aged 6–12 years, while adolescents were defined as those aged 13–18 years. Exclusion criteria included any comorbidity restricting participation in physical activity, such as cardiovascular disease or severe obesity; insufficient understanding of the Spanish language, and being in the honeymoon phase, defined as having insulin requirements ≤0.5 U/kg/day and an HbA_1c_ level ≤6% [25]. Of 183 patients assessed in the Paediatric Endocrinology Unit, 142 met the eligibility criteria, and 83 consented to participate, leading to a 58% participation rate (Fig. 1). Although the sample was not randomly selected and may not be representative of all individuals with type 1 diabetes, it reflects a real-world clinical population.

### Variables

#### Socioeconomic status

Socioeconomic status was assessed using the Family Affluence Scale III (FAS-III; 0–13 points) [26], with higher scores indicating greater affluence.

#### Anthropometric parameters, peak height velocity and sex

Standing and sitting height were measured using a SECA123 stadiometer (Hamburg, Germany), with a standard wooden box for sitting on. Body weight was assessed in lightweight clothing using a SECA869 electronic scale.

The estimated years from peak height velocity (PHV) was used as a somatic maturational landmark. Moore’s equation, which incorporates height, sitting height and weight, together with sex and age [27], was used for its calculation. To provide more information about the sample, the maturational stages were categorised at each assessment point as follows: pre-pubertal (≤1 year from PHV), peri-pubertal (between −1 and 1 year from PHV) and post-pubertal (≥1 year from PHV) [28]. Sex was obtained from medical records.

#### Assessment of diabetes-related parameters

Medical records were used to collect information on disease duration and HbA_1c_ levels (mmol/mol), in accordance with patient data protection laws. Participants were invited to attend at the time of their annual blood test; if this did not coincide with the study timeline, they were invited to attend a separate blood draw. HbA_1c_ levels were analysed at the central laboratory of the hospital.

In addition, daily data on interstitial glucose (mmol/l), insulin doses (U/kg) and carbohydrate ratio were extracted from the software associated with the FreeStyle continuous glucose measurement devices (Abbott Diabetes Care) and MiniMed devices (Medtronic) during a 9 day period coinciding with the accelerometry measurements.

#### Daily 24 h movement behaviours

The volume and intensity of physical activity, sedentary time and sleep duration were quantified using GENEActive triaxial accelerometer (ActiveInsights) worn on the wrist of the non-dominant hand for 9 days. This device was selected for its capacity to provide raw acceleration data, enabling flexible data processing [29], and its prior use in studies involving this population [30]. The pre-recording configuration was set at 85.7 Hz for nine consecutive days [31], starting at 20:00 hours on the day of evaluation. Data collected during the first night were used for calibration. GENEActiv PC software version 3.3 was used to collect accelerometer data, and the GGIR package was used to process the information [29].

To ensure a robust analysis, a minimum of 7 days of complete daily movement data, including at least one weekend day, was required for each of the three evaluations. The device was required to be worn for the entire 24 h period, including during sleep time. In addition, to understand the daily movement behaviour of the participants, the accelerometric recordings were complemented by a diary in which participants documented when and why they removed their wrist device if they did. To classify physical activity into specific intensity categories, age-specific cut-off points recommended by the GGIR package were applied separately for children and adolescents [32, 33]: physical inactivity time (0–56.3 milli-gravitational units [mG] for children; 0–50 mG for adolescents), light physical activity (LIPA, 56.3–191.6 mG for children; 50–150 mG for adolescents), moderate physical activity (191.6–695.8 mG for children; 150–500 mG for adolescents) and vigorous physical activity (>695.8 mG for children; >500 mG for adolescents). The duration of sleep was also determined as described by van Hees et al [34]. Periods of sustained inactivity, defined as those in which the arm angle did not change by more than 5° for at least five consecutive minutes, were identified as potential sleep episodes. To ensure the detection of nocturnal sleep and distinguish it from other periods of inactivity, the self-reported diary was used as a reference. After processing the raw accelerometer data, actual sleep duration was estimated, excluding periods when participants were in bed with the intention of sleeping but awake. Finally, sedentary behaviour was defined as any waking activity with an energy expenditure ≤1.5 metabolic equivalents of task in a sitting, reclining or lying posture.

### Statistical methods

All analyses were performed using R software for statistical computing version 4.4.1 (R Development Core Team, Vienna, Austria), through the RStudio interface (version 2024.09.0+375; Posit, Boston, MA, USA). Statistical significance was set at the conventional 0.05 level in all analyses.

#### Normality and descriptive statistics

Density plots and quantile–quantile plots were used to evaluate the normality of variable distributions, and the Shapiro–Wilk test was used to confirm. Descriptive statistics are reported as counts and percentages (%) for categorical variables, means ± SD for normally distributed continuous variables, and medians with IQRs for non-normally distributed continuous variables.

#### Differences between baseline and follow-up measurements

Paired *t* tests or the Wilcoxon signed-rank test were used to assess differences between baseline and the second follow-up. For categorical variables, proportions were compared. Differences between participants who remained in the study and those who withdrew were analysed using independent *t* tests or Mann–Whitney *U* tests. Similar tests were used to compare HbA_1c_ and interstitial glucose levels by sex.

#### Compositional data analysis

Compositional data analysis was applied to evaluate the relationships between movement behaviours (e.g. sedentary behaviour, LIPA, MVPA and sleep) and glycaemic outcomes (HbA_1c_ and interstitial glucose). This approach considers the co-dependent, relative nature of 24 h time use and addresses multicollinearity [35, 36]. A variance matrix was used to summarise data variability, with lower values indicating greater interdependence between behaviour pairs. In addition, the R packages ‘ggtern’ and ‘ggplot2’ were used in the creation of ternary plots with concentric iso-distance curves to represent the distribution of the compositions in the three assessment time points.

#### Isometric log-ratio transformations

The variables were mapped into Euclidean space by transforming them, via the R package ‘compositions’, into isometric log-ratio coordinates (*ilr*), representing the effect of increasing one behaviour at the proportional expense of the others, respecting the theoretical time frame of 24 h. This approach preserves the inherent relationships between variables.

To obtain behaviour-specific estimates, we constructed separate sets of *ilr* coordinates (*Z*) for each pattern by rotating the reference component in the transformation (e.g. *Z*_LIPA_, *Z*_MVPA_, *Z*_sedentary behaviour_ and *Z*_sleep_). This method highlights the relative contribution of the target behaviour through the first *ilr* coordinate (see electronic supplementary material [ESM] Table 1).

#### Linear mixed models

Linear mixed models were applied using the R package ‘lme4’ to estimate unstandardised beta coefficients (*B*) and 95% confidence interval (CI) for the relationship of each *ilr* composition set (*Z*) in separate models (i.e. independent variables) with HbA_1c_ or interstitial glucose (i.e. dependent variables), as well as standardised beta coefficients (*β*) to assess the partial effect size of the predictors. Models were adjusted for the three repeated measures, PHV and disease duration, as well as daily insulin doses and carbohydrate ratio, if applicable. In addition, interindividual variability that was not explained by fixed effects was considered a random effect due to the possible heterogeneity of the time spent on the various behaviours [37, 38]. Socioeconomic status was excluded as a covariate as most participants came from medium-to-high socioeconomic backgrounds. In addition, all assumptions for each model were evaluated. The *p* value reflects the effect of reallocating time between behaviours within a fixed 24 h period, and therefore the individual behaviours should not be interpreted as independent predictors.

#### Missing data

The R package ‘naniar’ was used to study the missing data (2.9%), which were due to random reasons (‘missing at random’) (e.g. data loss due to accelerometer raw data processing or unspecified issues in glucose monitoring) [39]. However, no imputation was performed to preserve the 24 h compositional structure and avoid biasing any variable, as these models are inherently robust to missing data without introducing bias.

#### Power analysis

Statistical power was estimated using the simulation-based R package ‘mixedpower’ [40], which accounts for both fixed and random effects. Assuming a small to moderate effect size (*β*=0.20–0.30), the available sample size (*n*=83 at baseline, *n*=62 at final follow-up) yielded >80% power to detect associations at α=0.05. Model performance was tested using the conditional coefficient of determination (*R*_c_^2^) to quantify the total variance explained by fixed and random effects [41] and the intraclass correlation coefficient to assess general contextual effects [42].

#### Sensitivity and subgroup analyses

To explore the possible impact of attrition-related selection bias, the main analysis was replicated in the subsample of individuals who completed the study. Sensitivity analyses were also performed to assess the impact of modelling the year of measurement as a categorical variable rather than a continuous variable. Additional models examined interactions with year of measurement for HbA_1c_ and interstitial glucose. Due to collinearity, alternative models included age and sex in place of PHV. Cross-sectional regressions were performed at each time point, and models with Δ*ilr* coordinates were analysed to explore within-patient variation. LIPA and MVPA were also grouped as a combined physical activity variable in sensitivity models.

## Results

### Main characteristics

In this study, 46 (55%) boys and 37 (45%) girls participated, with a median age of 13 years (IQR 11.00–15.00) and a disease duration of 4.08 years (IQR 2.01–7.03). The mean HbA_1c_ was 57.54 ± 9.22 mmol/mol (7.4 ± 0.8%), and the median interstitial glucose level was 9.37 mmol/l (IQR 8.68–10.31). At baseline, no significant differences were found between sexes for HbA_1c_ (boys: 57.54 mmol/mol [7.4%]; girls: 57.55 mmol/mol [7.4%]; *p*=0.995) and interstitial glucose levels (boys: 9.62 mmol/l; girls: 9.94 mmol/l; *p*=0.451). As shown in Fig. 1, of the total sample, 64 participants completed the first year of follow-up and 62 completed the second year, representing dropout rates of 23% and 25%, respectively, due to voluntary withdrawal. There were no missing values for any of the variables of interest at baseline, but glucose values were missing for one patient (1.6%) at the first follow-up and four patients (6.5%) at the second follow-up.

Descriptive characteristics for the patients at each measurement point, as well as comparisons between baseline and second follow-up, are presented in Table 1. Significant differences were found for all variables except sex, socioeconomic status, interstitial glucose and daily carbohydrate intake. Table 2 presents the variance matrix of the pairwise log ratios at each assessment, which describes the relative dispersion of the compositional components within the restricted 24 h time frame. Higher variance values reflect greater heterogeneity in the proportional distribution of time between behaviours, whereas lower values indicate more stable relative relationships between components. Differences between participants who completed the study and those who dropped out are reported in ESM Table 2; significant differences were found only for LIPA (*p*=0.048).

**Table 1:** Baseline and follow-up participant characteristics for the Diactive-1 cohort study

Variable	At baseline (*n*=83)	At 1-year follow-up (*n*=64)	At 2-year follow-up (*n*=62)	*p* value
Age (years)	13 (11.00–15.00)	14 (11–16)	15 (12–17)	<0.001^***^
Children	35 (42)	24 (37)	16 (26)	0.002^*^
Adolescents	48 (58)	40 (63)	46 (74)	0.002^*^
Diabetes duration (years)	4.08 (2.01–7.03)	5.08 (3.01–8.03)	6.08 (4.01–9.03)	<0.001^***^
Sex				
Boys	46 (55)	33 (52)	32 (52)	0.774
Girls	37 (45)	31 (48)	30 (48)	0.774
Socioeconomic status	8.00 (7–9)	9.00 (8–10)	9.00 (7–10)	0.328
High	17 (21)	18 (28)	20 (32)	0.246
Medium	53 (64)	39 (61)	32 (52)	<0.001^***^
Low	13 (16)	7 (11)	10 (16)	0.203
Maturation				
PHV (score)	−0.45 ± 1.91	0.16 ± 1.87	0.74 ± 1.79	<0.001^***^
Pre-pubertal	30 (36)	20 (31)	10 (16)	<0.001^***^
Peri-pubertal	34 (41)	19 (30)	22 (35)	0.020^*^
Post-pubertal	19 (23)	25 (39)	30 (48)	0.008^**^
Diabetes-related assessments				
HbA_1c_ (mmol/mol)	57.54 ± 9.22	58.12 ± 12.11	61.57 ± 14.13	0.003^**^
HbA_1c_ (%)	7.4 ± 0.8	7.4 ± 1.1	7.7 ± 1.2	
Interstitial glucose (mmol/l)	9.37 (8.68–10.31)	9.33 (8.41–10.63)	9.68 (8.49–10.78)	0.105
Daily insulin dose (U/kg)	0.69 (0.61–0.91)	0.77 (0.61–0.94)	0.85 (0.73–1.02)	0.025^*^
Daily carbohydrate intake (ratio)	16.62 ± 4.89	16.74 ± 6.12	15.68 ± 6.41	0.292
Physical activity				
LIPA (min)	240.03 ± 47.80	244.79 ± 52.70	227.65 ± 53.98	0.002^**^
MVPA (min)	81.22 (63.19–107.09)	70.24 (55.34–96.72)	72.82 (50.75–97.48)	0.001^**^
Total PA (min)	332.13 ± 81.18	329.35 ± 81.89	303.53 ± 80.97	0.001^**^
Sedentary behaviour (min)	676.99 ± 91.68	686.23 ± 101.64^a^	740.25 ± 99.50	<0.001^***^
Sleep time (min)	430.69 ± 43.07	426.10 ± 43.53	396.20 ± 52.44	<0.001^***^
24 h transformed variables^a^				
LIPA (min)	235.16	241.98	220.17	
MVPA (min)	83.42	72.81	69.41	
Total PA (min)	318.68	314.79	289.58	
Sedentary behaviour (min)	670.27	676.27	733.45	
Sleep time (min)	428.47	424.17	392.72	

Values are presented as medians with IQR for non-normally distributed continuous variables, means ± SD for normally distributed continuous variables, and *n* (%) for categorical variables

^a^Geometric means

^*^p<0.05, ^**^p<0.005 and ^***^p<0.001 obtained using the paired proportions comparison test or the Wilcoxon or paired *t* test between baseline and 2-year follow-up

PA, physical activity

**Table 2:** Variation matrix for daily movement behaviours

	LIPA	MVPA	Sedentary behaviour	Sleep
At baseline (*n*=83)				
LIPA	0			
MVPA	0.138	0		
Sedentary behaviour	0.107	0.331	0	
Sleep	0.055	0.224	0.435	0
At 1-year follow-up (*n*=64)				
LIPA	0			
MVPA	0.159	0		
Sedentary behaviour	0.122	0.380	0	
Sleep	0.047	0.237	0.055	0
At 2-year follow-up (*n*=62)				
LIPA	0			
MVPA	0.124	0		
Sedentary behaviour	0.131	0.337	0	
Sleep	0.074	0.232	0.058	0

**Fig. 2:**
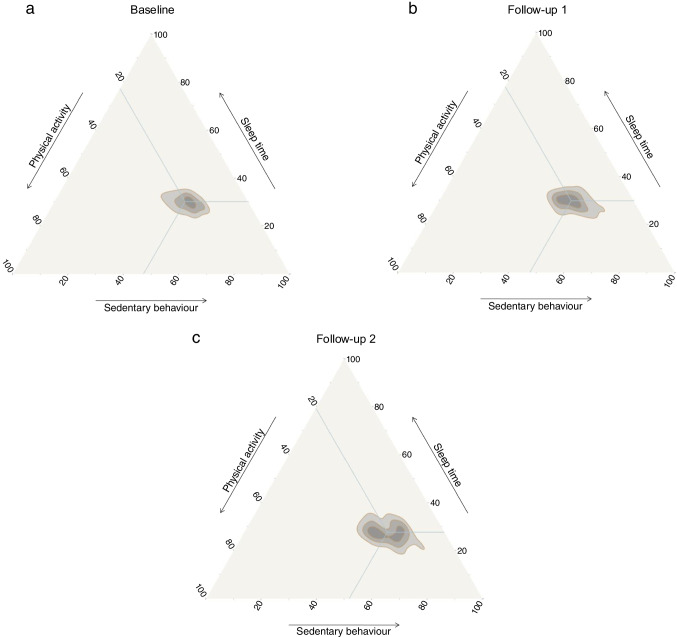
Ternary plots for daily time use in movement behaviours at three time points: (**a**) baseline; (**b**) first year of follow-up; (**c**): second year of follow-up. Blue lines converge to the geometric mean for the respective time point and movement behaviour (see Table 1)

Figure 2 illustrates the relative distribution of the three main components of the 24 h movement behaviour in this cohort. The plots reflect a proportional shift towards increased sedentary behaviour over time, relative to physical activity and sleep, across the three assessment points. ESM Fig. 1 illustrates the average time spent in each movement pattern across the three assessment time points (baseline, follow-up 1, and follow-up 2), with sedentary behaviour consistently occupying the largest proportion of time. At baseline, the geometric means for total physical activity, sedentary behaviour and sleep were 318.68, 670.27 and 428.47 min, respectively, per day. For the first follow-up, these values were 314.79 min of physical activity, 676.27 min of sedentary behaviour and 424.17 min of sleep. For the second follow-up, these values were 289.58 min of physical activity, 733.45 min of sedentary behaviour and 392.72 min of sleep (Table 1).

### Longitudinal associations

The results of the linear mixed models performed to analyse the associations between different sets of movement behaviour compositions with respect to HbA_1c_, considering time of measurement, PHV, disease duration and unexplained variability among participants, are presented in Table 3. Notably, increased daily sedentary behaviour at the expense of the other behaviours was associated with higher HbA_1c_ levels (*B*=14.077; 95% CI 4.244, 23.956; *β*=0.368). Increases in daily sleep time at the expense of the other habits was associated with lower HbA_1c_ levels (*B*=−12.712; 95% CI −25.204, −0.520; *β*=−0.197). When analysing other more specific coordinates, significant associations were found with the increase in the daily amount of MVPA (*B*=−9.361; 95% CI −15.856, −2.881; *β*=−0.330) or LIPA (*B*=−8.715; 95% CI −17.206, −0.104; *β*=−0.180) and decreasing duration of sedentary behaviour.

**Table 3:** Results of multilevel composition models for LIPA, MVPA, sedentary behaviour and sleep in relation to HbA_1c_

Fixed effect	*B*	95% CI	*β*	*p* value
*Z*_LIPA_				
*ilr*_1_	−0.155	−9.330, 9.258	−0.002	0.973
*ilr*_2_	−1.337	−5.742, 3.145	−0.048	0.556
*ilr*_3_	−16.404	−29.442, −3.584	−0.231	0.014^*^
*Z*_MVPA_				
*ilr*_1_	−1.209	−6.542, 4.140	−0.040	0.661
*ilr*_2_	−13.910	−27.173, −0.970	−0.155	0.038^*^
*ilr*_3_	−8.715	−17.206, −0.104	−0.180	0.048^*^
*Z*_sedentary behaviour_				
*ilr*_1_	14.077	4.244, 23.956	0.368	0.006^**^
*ilr*_2_	3.694	−3.311, 10.729	0.104	0.309
*ilr*_3_	7.689	−3.403, 19.124	0.109	0.179
*Z*_sleep_				
*ilr*_1_	−12.712	−25.204, −0.520	−0.197	0.044^*^
*ilr*_2_	−4.659	−13.471, 4.290	−0.067	0.308
*ilr*_3_	−9.361	−15.856, −2.881	−0.330	0.005^**^
PHV	−1.054	−2.444, 0.386	−0.170	0.133
Diabetes duration	0.187	−0.505, 0.889	0.057	0.588
Year of measurement	1.395	−0.129, 3.204	0.097	0.088
Random effects: participants	8.982^a^		80.67^b^	
Model performance	0.610^c^		0.587^d^	

The *Z*_LIPA_ model is based on the set (*Z*) of coordinates (*ilr*) for LIPA: *ilr*_1_ (LIPA at the expense of MVPA, sedentary behaviour and sleep), *ilr*_2_ (MVPA at the expense of sleep and sedentary behaviour) and *ilr*_3_ (sleep at the expense of sedentary behaviour)

The *Z*_MVPA_ model is based on the set (*Z*) of coordinates (*ilr*) for MVPA: *ilr*_1_ (MVPA at the expense of LIPA, sedentary behaviour and sleep), *ilr*_2_ (sleep at the expense of LIPA and sedentary behaviour) and *ilr*_3_ (LIPA at the expense of sedentary behaviour)

The *Z*_sedentary behaviour_ model is based on the set (*Z*) of coordinates (*ilr*) for sedentary behaviour: *ilr*_1_ (sedentary behaviour at the expense of LIPA, MVPA and sleep), *ilr*_2_ (MVPA at the expense of LIPA and sleep) and *ilr*_3_ (LIPA at the expense of sleep)

The *Z*_sleep_ model is based on the set (*Z*) of coordinates (*ilr*) for sleep: *ilr*_1_ (sleep at the expense of LIPA, MVPA and sedentary behaviour), *ilr*_2_ (LIPA at the expense of MVPA and sedentary behaviour) and *ilr*_3_ (MVPA at the expense of sedentary behaviour)

Models were adjusted for each set (*Z*) of coordinates (*ilr*), PHV, disease duration, year of measurement (fixed effects) and participants identification (random effect)

^a^SD; ^b^variance; ^c^*R*_c_^2^ (conditional coefficient of determination); ^d^intraclass correlation coefficient

^*^p<0.05, ^**^p<0.005

Table 4 shows the associations between the various sets of movement patterns with respect to interstitial glucose. Greater interstitial glucose levels were associated with increased daily sedentary time compared with the other behaviours (*B*=1.988, 95%CI 0.153 to 3.880, *β*=0.261). Furthermore, spending more daily time in MVPA (*B*=−1.580; 95% CI −2.800, −0.388; *β*=−0.283) or increasing sleep duration (*B*=−0.670; 95% CI −5.230, −0.196; *β*=−0.176) at the expense of sedentary behaviour is associated with reductions in interstitial glucose.

**Table 4:** Results of multilevel composition models for LIPA, MVPA, sedentary behaviour and sleep in relation to interstitial glucose

Fixed effect	*B*	95% CI	*β*	*p* value
*Z*_LIPA_				
*ilr*_1_	0.978	−0.640, 2.620	0.068	0.248
*ilr*_2_	−0.289	−1.150, 0.584	−0.052	0.516
*ilr*_3_	−0.670	−5.230, −0.196	−0.176	0.038^*^
*Z*_MVPA_				
*ilr*_1_	−0.598	−1.550, 0.369	−0.102	0.232
*ilr*_2_	−2.720	−5.320, −0.214	−0.133	0.037^*^
*ilr*_3_	−0.619	−2.140, 0.881	−0.064	0.430
*Z*_sedentary behaviour_				
*ilr*_1_	1.988	0.153, 3.880	0.261	0.038^*^
*ilr*_2_	0.068	−1.210, 1.370	0.009	0.917
*ilr*_3_	2.049	−0.061, 4.230	0.139	0.062
*Z*_sleep_				
*ilr*_1_	−2.370	−4.850, 0.022	−0.171	0.056
*ilr*_2_	0.200	−1.330, 1.730	0.014	0.802
*ilr*_3_	−1.580	−2.800, −0.388	−0.283	0.012^*^
PHV	−0.149	−0.407, 0.108	−0.115	0.268
Diabetes duration	−0.025	−0.158, 0.106	−0.036	0.707
Year of measurement	0.007	−0.293, 0.306	0.002	0.959
Daily insulin doses	3.290	2.010, 4.580	0.366	<0.001^**^
Carbohydrate ratio	−0.060	−2.014, −0.185	−0.142	0.021^*^
Random effects				
Participants	1.73^a^		2.99^b^	
Model performance	0.740^c^		0.673^d^	

The *Z*_LIPA_ model is based on the set (*Z*) of coordinates (*ilr*) for LIPA: *ilr*_1_ (LIPA at the expense of MVPA, sedentary behaviour and sleep), *ilr*_2_ (MVPA at the expense of sleep and sedentary behaviour) and *ilr*_3_ (sleep at the expense of sedentary behaviour)

The *Z*_MVPA_ model is based on the set (*Z*) of coordinates (*ilr*) for MVPA: *ilr*_1_ (MVPA at the expense of LIPA, sedentary behaviour and sleep), *ilr*_2_ (sleep at the expense of LIPA and sedentary behaviour) and *ilr*_3_ (LIPA at the expense of sedentary behaviour)

The *Z*_SB_ model is based on the set (*Z*) of coordinates (*ilr*) for sedentary behaviour: *ilr*_1_ (sedentary behaviour at the expense of LIPA, MVPA and sleep), *ilr*_2_ (MVPA at the expense of LIPA and sleep) and *ilr*_3_ (LIPA at the expense of sleep)

The *Z*_sleep_ model is based on the set (*Z*) of coordinates (*ilr*) for sleep: *ilr*_1_ (sleep at the expense of LIPA, MVPA and sedentary behaviour), *ilr*_2_ (LIPA at the expense of MVPA and sedentary behaviour) and *ilr*_3_ (MVPA at the expense of sedentary behaviour)

Models were adjusted for each set (*Z*) of coordinates (*ilr*), PHV, disease duration, year of measurement, daily insulin doses, carbohydrate intake ratio (fixed effects) and participants identification (random effect)

^a^SD; ^b^variance; ^c^*R*_c_^2^ (conditional coefficient of determination); ^d^intraclass correlation coefficient

^*^p<0.05, ^**^p<0.001

ESM Table 3 presents the results when total physical activity (i.e. MVPA and LIPA combined) is considered as a single category. Sedentary behaviour at the expense of physical activity and sleep (*B*=15.062; 95% CI 4.709. 25.476; *β*=0.305), or sleep at the expense of physical activity and sedentary behaviour (*B*=−13.122; 95% CI −26.185, −0.345; *β*=−0.142) were associated with HbA_1c_ levels. When analysing other more specific coordinates, significant associations were found with increasing physical activity time (*B*=−9.998; 95% CI −16.783, −2.776; *β*=−0.234) and decreasing daily time in sedentary behaviour. ESM Table 4 presents analogous analyses focusing on interstitial glucose, which yielded results consistent with the directionality of the results reported above for HbA_1c_.

Finally, when including year of measurement as a factor, the results for HbA_1c_ remained similar (ESM Table 5), although an increased proportion of daily sleep was associated with lower interstitial glucose (*B*=−2.691; 95% CI −5.145, −0.290; *β*=−0.194) (ESM Table 6). No significant interactions with time between measurements were observed in any of the models studied. Moreover, models adjusted for age and sex (ESM Tables 7 and 8), cross-sectional models at each assessment (ESM Tables 9 and 10), and models using Δ*ilr* coordinates (ESM Tables 11 and 12) yielded consistent directionality of associations. Results restricted to participants who completed both follow-up evaluations (*n*=62) were also in line with the main findings (ESM Tables 13 and 14).

## Discussion

The main findings of this study indicate that, in young people with type 1 diabetes, more sleep time relative to other behaviours is associated with lower HbA_1c_ levels, whereas increased daily sedentary behaviour relative to other behaviours is associated with poorer metabolic stability and higher interstitial glucose; highlighting the novel role of sleep compared with the detrimental character of prolonged sedentarism. Furthermore, increases in MVPA at the expense of reducing sedentary behaviour also show inverse associations with HbA_1c_ and interstitial glucose. These findings are in line with current guidelines [13], which advocate regular physical activity and a reduction of sedentary time [9, 12]. However, it is clinically relevant to approach movement behaviours holistically, considering the balance between movement behaviours across the 24 h day [24], as other studies in apparently healthy young people without diabetes have demonstrated [43]. This integrated perspective may offer a clearer understanding of how movement patterns interact with glycaemic dynamics and consequent HbA_1c_ levels. Despite these results, at each time point, participants had metabolic stability levels close to the ADA recommended targets [10], probably due to regular follow-up by specialised paediatric endocrinologists and the use of diabetes technologies, resulting in a relatively well-controlled glycaemic profile that should be considered when interpreting the findings and generalising them to other populations.

Within 24 h movement behaviours, young people with type 1 diabetes tend to report shorter sleep durations compared with their healthy peers [44], probably due to disease management factors that can disrupt sleep patterns [45]. Variations in sleep have been linked to HbA_1c_ levels [46], and higher HbA_1c_ has been observed in those with shorter sleep durations [20]. Cross-sectional research further indicates that those with shorter sleep durations tend to exhibit higher HbA_1c_ levels [47] and elevated glucose levels [22]. Our findings align with these results; however, by employing a compositional approach coupled with a longitudinal design, we provide a deeper understanding. A possible physiological explanation may revolve around insulin sensitivity and the resulting reduction in glucose levels. Adequate sleep patterns help to regulate cortisol levels, promote growth hormone secretion [44] and even regulate the concentration of anti-inflammatory cytokines [20].

Previous research has also reported that young people with type 1 diabetes spend 63.3 min more in sedentary behaviour compared with their healthy peers [15]. This significant amount of sedentary behaviour may partially explain the variance in HbA_1c_ due to the positive association between both variables [48], which reinforces our longitudinal compositional findings and is in line with the ADA recommendations that advocate reducing sedentary time to avoid glycaemic imbalances [9]. Additionally, whereas previous research has related higher levels of physical activity, as an independent predictor, to lower HbA_1c_ [48], our findings showed no significant association with either HbA_1c_ or interstitial glucose when total physical activity was increased at the expense of sedentary behaviour and/or sleep. This may be due to the role of adequate sleep time or the importance of the type of physical activity intensity, as time spent on MVPA appeared to be more relevant than that spent on LIPA in our results. However, significant associations were found for HbA_1c_ when total physical activity or MVPA increased specifically at the expense of sedentary behaviour, and for glycaemic values when MVPA increased at the expense of sedentarism. This latter finding is consistent with other studies that reported that MVPA has an emerging role as a predictor of glycaemic stability [17]. The absence of a significant association when sleep time is included in the formula may be explained by the need to maintain adequate sleep time to ensure optimal metabolic balance as explained above. This underlines the importance of sleep time on HbA_1c_ and glucose levels, highlighting the need to take 24 h movement patterns into account in future clinical strategies.

To our knowledge, this is the first study in children and adolescents with type 1 diabetes to associate movement behaviours and HbA_1c_ levels using a 24 h approach. This study emphasises the health implications of the interaction and dependence between daily behavioural variables rather than their absolute durations. A key strength of the study is the use of linear mixed regression models within a longitudinal framework. This approach considers both repeated observations for each participant and unexplained variability due to heterogeneity in how individuals adjust their daily activities. However, there are some limitations that should be discussed. First, accelerometers are unable to capture all aspects of everyday behaviours, such as the specific type of activity performed (e.g. cycling). The small sample size, wide age range and participant withdrawals limited the statistical power, hampering subgroup and interaction analyses and limiting generalisability. Despite adjusting for maturation, developmental differences in sleep needs may influence associations with glycaemic stability. Furthermore, while missing data were handled using a structured approach, the possibility that they follow an MNAR pattern (missing not at random) cannot be entirely ruled out.

Our analyses were adjusted for both age and sex to account for potential confounding effects, and the results remained stable after adjustment. Although sex-stratified analyses were not conducted, adjusting for sex ensures that the associations observed are not driven by sex differences. Given this adjustment, the findings may be generalised to both boys and girls within similar clinical contexts.

In conclusion, healthcare professionals are encouraged to emphasise not only the promotion of physical activity and the reduction of sedentary behaviour, but also the importance of adequate sleep in children and adolescents with type 1 diabetes, considering movement behaviours within an integrated 24 h clinical framework. As increasing sleep and reducing sedentary behaviour in favour of physical activity may lower HbA_1c_ and glucose levels in young people with type 1 diabetes, we suggest that strategies such as educational programmes for sleep management and active breaks during prolonged sedentary periods may be effective, and should be considered in future research. Additionally, planning of physical activity tailored to individual preferences and needs may further enhance these outcomes. Future research should comprise randomised controlled trials evaluating integrated 24 h movement behaviour interventions and their impact on glycaemic stability as a non-pharmacological therapy [49], using technologies that capture the context and type of activity.

## Supplementary Information

Below is the link to the electronic supplementary material.

ESM (PDF 615 KB)

## Data Availability

The data are not publicly available because of privacy or ethical restrictions but are available upon request from the corresponding author.

## Abbreviations

LIPA: Light physical activity
*ilr*: Isometric log-ratio coordinates
MVPA: Moderate-to-vigorous physical activity
PHV: Peak height velocity
