# Supplementary material for: Compositional analysis of the association between 24 h movement behaviours, HbA1c and interstitial glucose in children and adolescents with type 1 diabetes mellitus: a two-year longitudinal analysis of the Diactive-1 cohort study
Source: Diabetologia. 2025 Jul 31;68(10):2126–38. doi: 10.1007/s00125-025-06496-2 (PMC12423199; doi:10.1007/s00125-025-06496-2)

Electronic supplementary material (ESM) for:

Compositional analysis of the association between 24 h movement behaviours, HbA_{1c} and interstitial glucose in children and adolescents with type 1 diabetes mellitus: a two-year longitudinal analysis of the Diactive-1 cohort study

Jacinto Muñoz-Pardeza; José Francisco López-Gil; Ignacio Hormazábal-Aguayo; Nidia Huerta-Uribe; Yasmin Ezzatvar; Antonio García-Hermoso

Table of contents

ESM Table 1. 2
ESM Table 2. 3
ESM Table 3. 4
ESM Table 4. 5
ESM Table 5. 6
ESM Table 6. 7
ESM Table 7. 8
ESM Table 8. 9
ESM Table 9. 10
ESM Table 10. 11
ESM Table 11. 12
ESM Table 12. 13
ESM Table 13. 14
ESM Table 14. 15
ESM Fig. 1. 16

**** Corresponding author***
Antonio García-Hermoso
antonio.garciah@unavarra.es

ESM Table 1. *Formulas used to calculate each set of log-ratio isometric coordinate compositions of the daily behavioural variables.*

**Z= Light intensity physical activity**	
ilr_1	$= \sqrt{\frac{3}{4}} ln \frac{LIPA}{(MVPA \cdot Sleep \cdot SB)^{1/3}}$
ilr_2	$= \sqrt{\frac{2}{3}} ln \frac{MVPA}{(Sleep \cdot SB)^{1/2}}$
ilr_3	$= \sqrt{\frac{1}{2}} ln \frac{Sleep}{SB}$
**Z= Moderate-vigorous physical activity**	
ilr_1	$= \sqrt{\frac{3}{4}} ln \frac{MVPA}{(LIPA \cdot Sleep \cdot SB)^{1/3}}$
ilr_2	$= \sqrt{\frac{2}{3}} ln \frac{Sleep}{(LIPA \cdot SB)^{1/2}}$
ilr_3	$= \sqrt{\frac{1}{2}} ln \frac{LIPA}{SB}$
**Z= Sedentary behaviour**	
ilr_1	$= \sqrt{\frac{3}{4}} ln \frac{SB}{(LIPA \cdot MVPA \cdot Sleep)^{1/3}}$
ilr_2	$= \sqrt{\frac{2}{3}} ln \frac{MVPA}{(LIPA \cdot Sleep)^{1/2}}$
ilr_3	$= \sqrt{\frac{1}{2}} ln \frac{LIPA}{Sleep}$
**Z= Sleep time**	
ilr_1	$= \sqrt{\frac{3}{4}} ln \frac{Sleep}{(LIPA \cdot MVPA \cdot SB)^{1/3}}$
ilr_2	$= \sqrt{\frac{2}{3}} ln \frac{LIPA}{(MVPA \cdot SB)^{1/2}}$
ilr_3	$= \sqrt{\frac{1}{2}} ln \frac{MVPA}{SB}$

Z: Compositions for each variable; *Ilr*: Log-ratio isometric coordinate; LIPA: Light intensity physical activity; MVPA: Moderate-vigorous physical activity; SB: Sedentary behaviour.

ESM Table 2. *Differences between those who left the study and those who continued the 1st and 2nd year follow-up.*

Variables	Lost to follow-up (n = 21)	Followed 1st and 2nd year. (n = 62)	p value
Age (years) [a]	15 (3)	13 (5)	0.200
Children	8 (38)	27 (44)	0.331
Adolescents	13 (62)	35 (56)	0.331
Diabetes Duration (years) [a]	4.47 (4.35)	3.84 (5.21)	0.258
Sex			
Boys	14 (67)	32 (52)	0.115
Girls	7 (33)	30 (48)	0.115
Socioeconomic status [a]	9.00 (2.00)	8.00 (2)	0.653
High level	2 (9)	15 (24)	0.075
Medium level	14 (67)	39 (63)	0.378
Low level	5 (24)	8 (13)	0.117
**Maduration**			
Peak Height Velocity (score)	-0.31 ± 2.12	-0.51± 1.83	0.683
Pre-pubertal	7 (33)	23 (37)	0.378
Peri-pubertal	9 (43)	25 (40)	0.419
Post-pubertal	5 (24)	14 (23)	0.454
**Diabetes related-assessment**			
HbA_{1c} (mmol/mol)	59.230 ± 8.397	56.903 ± 9.509	0.282
HbA_{1c} (%)	7.5 ± 0.7	7.3 ± 0.8	
Interstitial glucose (mmol/l) [a]	9.68 (1.95)	9.37 (2.14)	0.513
Daily insulin dose (U/kg) [a]	0.68 (0.30)	0.71 (0.31)	0.904
Daily carbohydrate intake (R)	17.11 ± 5.47	16.48 ± 4.75	0.667
**Physical Activity**			
Light intensity PA (min)	222.09 ± 48.76	246.61 ± 46.11	0.048*
Moderate-vigorous PA (min) [a]	83.70 (44.07)	80.59 (43.59)	0.850
Total PA (min)	307.68 ± 77.43	341.10 ± 81.30	0.095
Sedentary behaviour (min)	705.82 ± 81.47	666.24 ± 93.58	0.068
Sleep time (min)	427.10 ± 47.98	432.03 ± 41.46	0.672

[a] Values are presented as: medians with interquartile range for non-normally distributed continuous variables.

Other values are presented as: means ± SD for normally distributed continuous variables, and n (%) for categorical variables.

*$p<0.05$ obtained using the paired proportions comparison test or the Mann-Whitney U test or t-test for differences between those who left the study and those who continued the 1st and 2nd year follow-up.

HbA_{1c}: Glycated Haemoglobin; PA: Physical activity.

ESM Table 3. *Results from multilevel composition models for physical activity, sedentary behaviour and sleep in relation to HbA_{1c}.*

Fixed effects	*B*	95% CI	β	*p* value
Z_{PA}				
ilr_1	-2.255	-9.319, 5.033	-0.049	0.533
ilr_2	-16.090	-29.136, -3.252	-0.227	0.016^{*}
Z_{SB}				
ilr_1	15.062	4.709, 25.476	0.305	0.005^{**}
ilr_2	6.091	-4.311, 16.779	0.098	0.255
Z_{sleep}				
ilr_1	-13.122	-26.185, -0.345	-0.142	0.049^{*}
ilr_2	-9.998	-16.783, -2.776	-0.234	0.006^{*}
PHV	-1.035	-2.371, 0.301	-0.167	0.135
Diabetes duration	0.179	-0.489, 0.850	0.054	0.603
Year of measurement	1.411	-0.175, 2.986	0.098	0.084
Random effects				
Participants	8.986 [a]		80.75 [b]	
Model performance	0.612 [c]		0.590 [d]	

Model based on the set (Z) of coordinates (ilr) for PA: $ilr^1 = \sqrt{\frac{2}{3}}\, ln \frac{PA}{(Sleep \cdot SB)^{1/2}}$ (PA at the expense of SB, and sleep), $ilr^2 = \sqrt{\frac{1}{2}}\, ln \frac{Sleep}{SB}$ (Sleep at the expense of SB).

Model based on the set (Z) of coordinates (ilr) for SB: $ilr^1 = \sqrt{\frac{2}{3}}\, ln \frac{SB}{(PA \cdot Sleep)^{1/2}}$ (SB at the expense of PA, and sleep), $ilr^2 = \sqrt{\frac{1}{2}}\, ln \frac{PA}{Sleep}$ (PA at the expense of sleep).

Model based on the set (Z) of coordinates (ilr) for Sleep: $ilr^1 = \sqrt{\frac{2}{3}}\, ln \frac{Sleep}{(PA \cdot SB)^{1/2}}$ (Sleep at the expense of PA, and SB), $ilr^2 = \sqrt{\frac{1}{2}}\, ln \frac{PA}{SB}$ (PA at the expense of sleep and SB).

Models: adjusted for each set (*Z*) of coordinates (*ilr*), PHV, disease duration, year of measurement (*fixed effects*) and participants identification (*random effect*).

[a]SD; [b]variance, [c]R_c^2 (conditional coefficient of determination); [d]intraclass correlation coefficient

$^{*}p<0.05$, $^{**}p<0.005$

HbA1c, glycated hemoglobin; PA: physical activity; SB: Sedentary behaviour; PHV: Peak Height Velocity; *B*: Unstandardised beta coefficient; CI: Confident Interval; SD: standard deviation; SD^2: variance; R_c^2, conditional coefficient of determination; ICC, intraclass correlation coefficient.

ESM Table 4. *Results from multilevel composition models for physical activity, sedentary behaviour and sleep in relation to interstitial glucose levels.*

Fixed effects	*B*	95% CI	β	*p* value
Z_{PA}				
ilr_1	0.058	-1.340, 1.500	0.006	0.935
ilr_2	-2.610	-5.190, -0.128	-0.172	0.043*
Z_{SB}				
ilr_1	2.230	0.286, 4.240	0.224	0.028*
ilr_2	1.360	-0.730, 3.520	0.106	0.208
Z_{sleep}				
ilr_1	-2.290	-4.930, 0.249	-0.111	0.082
ilr_2	-1.260	-2.570, 0.050	-0.148	0.066
PHV	-0.118	-0.373, 0.136	-0.091	0.373
Diabetes duration	-0.028	-0.161, 0.104	-0.040	0.680
Year of measurement	0.009	-0.293, 0.310	0.003	0.950
Daily insulin doses	3.350	2.060, 4.640	0.373	<0.001**
Carbohydrate rations	-0.065	-0.017, -0.015	-0.153	0.012*
Random effects				
Participants		1.72 [a]	2.98 [b]	
Model performance		0.735 [c]	0.671 [d]	

Model based on the set (Z) of coordinates (ilr) for PA: $ilr^1 = \sqrt{\frac{2}{3}}\, ln \frac{PA}{(Sleep \cdot SB)^{1/2}}$ (PA at the expense of SB, and sleep), $ilr^2 = \sqrt{\frac{1}{2}}\, ln \frac{Sleep}{SB}$ (Sleep at the expense of SB).

Model based on the set (Z) of coordinates (ilr) for SB: $ilr^1 = \sqrt{\frac{2}{3}}\, ln \frac{SB}{(PA \cdot Sleep)^{1/2}}$ (SB at the expense of PA, and sleep), $ilr^2 = \sqrt{\frac{1}{2}}\, ln \frac{PA}{Sleep}$ (PA at the expense of sleep).

Model based on the set (Z) of coordinates (ilr) for Sleep: $ilr^1 = \sqrt{\frac{2}{3}}\, ln \frac{Sleep}{(PA \cdot SB)^{1/2}}$ (Sleep at the expense of PA, and SB), $ilr^2 = \sqrt{\frac{1}{2}}\, ln \frac{PA}{SB}$ (PA at the expense of sleep and SB).

Models: adjusted for each set of coordinates (*ilr*), PHV, disease duration, year of measurement, daily insulin doses, carbohydrate intake rations (fixed effects), and participants identification (random effect).

[a]SD; [b]variance, [c]R_c^2 (conditional coefficient of determination); [d]intraclass correlation coefficient

*$p<0.05$, **$p<0.001$

PA: physical activity; SB: Sedentary behaviour; PHV: Peak Height Velocity; *B*: Unstandardised beta coefficient; CI: Confident Interval; SD: standard deviation; SD^2: variance; R_c^2, conditional coefficient of determination; ICC, intraclass correlation coefficient.

ESM Table 5. *Results of multilevel compositional models examining associations between daily movement behaviours and HbA_{1c}, including year of measurement as a categorical variable.*

Fixed effects	*B*	95% CI	β	*p* value
Z_{LIPA}				
ilr_1	0.252	-9.117, 9.804	0.003	0.958
ilr_2	-1.501	-5.997, 3.045	-0.054	0.517
ilr_3	-15.728	-29.146, -2.466	-0.222	0.023*
Z_{MVPA}				
ilr_1	-1.499	-7.008, 4.019	-0.050	0.600
ilr_2	-13.489	-26.886, -0.336	-0.150	0.048*
ilr_3	-8.091	-17.063, 0.968	-0.167	0.083
Z_{SB}				
ilr_1	13.465	3.211, 23.752	0.352	0.012*
ilr_2	3.170	-4.266, 10.623	0.090	0.412
ilr_3	7.636	-3.499, 19.041	0.108	0.184
Z_{sleep}				
ilr_1	-12.218	-24.917, 0.261	-0.190	0.059
ilr_2	-4.051	-13.303, 5.295	-0.058	0.398
ilr_3	-9.164	-15.721, -2.617	-0.323	0.007*
PHV	-1.028	-2.383, 0.324	-0.166	0.144
Diabetes duration	0.187	-0.480, 0.856	0.057	0.588
Baseline (ref)				
Follow-up 1	0.901	-1.930, 3.705	0.075	0.535
Follow up-2	2.853	-0.334, 6.022	0.238	0.084
Random effects				
Participants	8.969 [a]		80.440 [b]	
Model performance	0.607 [c]		0.585 [d]	

The Z_{LIPA} model is based on the set (*Z*) of coordinates (*ilr*) for LIPA: ilr_1 (LIPA at the expense of MVPA, SB and sleep), ilr_2 (MVPA at the expense of sleep and SB) and ilr_3 (sleep at the expense of SB)

The Z_{MVPA} model is based on the set (*Z*) of coordinates (*ilr*) for MVPA: ilr_1 (MVPA at the expense of LIPA, SB and sleep), ilr_2 (sleep at the expense of LIPA and SB) and ilr_3 (LIPA at the expense of SB)

The Z_{SB} model is based on the set (*Z*) of coordinates (*ilr*) for SB: ilr_1 (SB at the expense of LIPA, MVPA and sleep), ilr_2 (MVPA at the expense of LIPA and sleep) and ilr_3 (LIPA at the expense of sleep)

The Z_{Sleep} model is based on the set (*Z*) of coordinates (*ilr*) for sleep: ilr_1 (sleep at the expense of LIPA, MVPA and SB), ilr_2 (LIPA at the expense of MVPA and SB) and ilr_3 (MVPA at the expense of SB)

Models: adjusted for each set (*Z*) of coordinates (*ilr*), PHV, disease duration, year of measurement (*fixed effects*) and participants identification (*random effect*).

[a]SD; [b]variance, [c]R_c^2 (conditional coefficient of determination); [d]intraclass correlation coefficient

*$p<0.05$

HbA1c, glycated haemoglobin; LIPA: Light intensity physical activity; MVPA: Moderate-vigorous physical activity; SB: Sedentary behaviour; PHV: Peak Height Velocity; *B*: Unstandardized beta coefficient; *β:* Standardized beta coefficient; CI: Confident Interval; SD: standard deviation; SD^2: variance; R_c^2, conditional coefficient of determination; ICC, intraclass correlation coefficient.

ESM Table 6. *Results of multilevel compositional models examining associations between daily movement behaviours and interstitial glucose, including year of measurement as a categorical variable.*

Fixed effects	*B*	95% CI	β	*p* value
Z_{LIPA}				
ilr$_1$	0.653	-0.991, 2.315	0.045	0.448
ilr$_2$	-0.137	-1.004, 0.735	-0.025	0.760
ilr$_3$	-3.109	-5.666, -0.604	-0.205	0.017*
Z_{MVPA}				
ilr$_1$	-0.347	-1.337, 0.646	-0.059	0.503
ilr$_2$	-2.977	-5.543, -0.469	-0.145	0.023*
ilr$_3$	-1.060	-2.638, 0.508	-0.110	0.198
Z_{SB}				
ilr$_1$	2.385	0.509, 4.294	0.313	0.015
ilr$_2$	0.475	-0.871, 1.831	0.068	0.499
ilr$_3$	2.048	-0.046, 4.190	0.139	0.061
Z_{sleep}				
ilr$_1$	-2.691	-5.145, -0.290	-0.194	0.031*
ilr$_2$	-0.258	-1.859, 1.340	-0.018	0.757
ilr$_3$	-1.673	-2.881, -0.482	-0.298	0.008*
PHV	-0.164	-0.420, 0.091	-0.127	0.221
Diabetes duration	-0.021	-0.152, 0.110	-0.030	0.757
Baseline (ref)				
Follow-up 1	0.361	-0.138, 0.857	0.147	0.166
Follow up-2	-0.036	-0.634, 0.558	-0.014	0.907
Daily insulin doses	3.327	2.052, 4.605	0.370	< 0.001**
Carbohydrate rations	-0.064	-0.115, -0.014	-0.151	0.014*
Random effects				
Participants	1.730 [a]		2.994 [b]	
Model performance	0.744 [c]		0.677 [d]	

The Z_{LIPA} model is based on the set (*Z*) of coordinates (*ilr*) for LIPA: *ilr*$_1$ (LIPA at the expense of MVPA, SB and sleep), *ilr*$_2$ (MVPA at the expense of sleep and SB) and *ilr*$_3$ (sleep at the expense of SB)

The Z_{MVPA} model is based on the set (*Z*) of coordinates (*ilr*) for MVPA: *ilr*$_1$ (MVPA at the expense of LIPA, SB and sleep), *ilr*$_2$ (sleep at the expense of LIPA and SB) and *ilr*$_3$ (LIPA at the expense of SB)

The Z_{SB} model is based on the set (*Z*) of coordinates (*ilr*) for SB: *ilr*$_1$ (SB at the expense of LIPA, MVPA and sleep), *ilr*$_2$ (MVPA at the expense of LIPA and sleep) and *ilr*$_3$ (LIPA at the expense of sleep)

The Z_{Sleep} model is based on the set (*Z*) of coordinates (*ilr*) for sleep: *ilr*$_1$ (sleep at the expense of LIPA, MVPA and SB), *ilr*$_2$ (LIPA at the expense of MVPA and SB) and *ilr*$_3$ (MVPA at the expense of SB)

Models: adjusted for each set of coordinates (ilr), PHV, disease duration, year of measurement, daily insulin doses, carbohydrate intake rations (fixed effects), and participants identification (random effect).

[a]SD; [b]variance, [c]R_c^2 (conditional coefficient of determination); [d]intraclass correlation coefficient

*$p<0.05$, **$p<0.005$

LIPA: Light intensity physical activity; MVPA: Moderate-vigorous physical activity; SB: Sedentary behaviour; PHV: Peak Height Velocity; *B*: Unstandardized beta coefficient; *β:* Standardized beta coefficient ; CI: Confident Interval; SD: standard deviation; SD^2: variance; R_c^2, conditional coefficient of determination; ICC, intraclass correlation coefficient.

ESM Table 7. *Results of multilevel compositional models examining associations between daily movement behaviours and HbA_{1c}, adjusted for age and sex.*

Fixed effects	*B*	95% CI	β	*p* value
Z_{LIPA}				
ilr$_1$	-1.076	-10.404, 8.544	-0.014	0.824
ilr$_2$	-0.594	-5.153, 4.126	-0.021	0.802
ilr$_3$	-15.451	-28.882, -2.323	-0.218	0.024*
Z_{MVPA}				
ilr$_1$	-0.201	-5.815, 5.483	-0.006	0.945
ilr$_2$	-12.774	-26.184, 0.220	-0.142	0.060
ilr$_3$	-8.776	-17.726, 0.320	-0.182	0.060
Z_{SB}				
ilr$_1$	13.254	3.123, 23.456	0.347	0.012*
ilr$_2$	4.472	-3.121, 12.169	0.127	0.258
ilr$_3$	6.674	-4.330, 18.103	0.095	0.244
Z_{sleep}				
ilr$_1$	-11.976	-24.772, 0.403	-0.186	0.064
ilr$_2$	-5.376	-14.680, 4.087	-0.077	0.267
ilr$_3$	-8.240	-14.648, -1.826	-0.290	0.013*
Sex	0.905	-3.903, 5.711	0.037	0.718
Age	-0.463	-1.434, 0.508	-0.108	0.361
Diabetes duration	0.146	-0.536, 0.829	0.044	0.680
Year of measurement	1.336	-0.270, 2.929	0.093	0.108
Random effects				
Participants		9.055 [a]	81.990 [b]	
Model performance		0.611 [c]	0.589 [d]	

The Z_{LIPA} model is based on the set (*Z*) of coordinates (*ilr*) for LIPA: *ilr*$_1$ (LIPA at the expense of MVPA, SB and sleep), *ilr*$_2$ (MVPA at the expense of sleep and SB) and *ilr*$_3$ (sleep at the expense of SB)

The Z_{MVPA} model is based on the set (*Z*) of coordinates (*ilr*) for MVPA: *ilr*$_1$ (MVPA at the expense of LIPA, SB and sleep), *ilr*$_2$ (sleep at the expense of LIPA and SB) and *ilr*$_3$ (LIPA at the expense of SB)

The Z_{SB} model is based on the set (*Z*) of coordinates (*ilr*) for SB: *ilr*$_1$ (SB at the expense of LIPA, MVPA and sleep), *ilr*$_2$ (MVPA at the expense of LIPA and sleep) and *ilr*$_3$ (LIPA at the expense of sleep)

The Z_{Sleep} model is based on the set (*Z*) of coordinates (*ilr*) for sleep: *ilr*$_1$ (sleep at the expense of LIPA, MVPA and SB), *ilr*$_2$ (LIPA at the expense of MVPA and SB) and *ilr*$_3$ (MVPA at the expense of SB)

Models: adjusted for each set (*Z*) of coordinates (*ilr*), sex, age, disease duration, year of measurement (*fixed effects*) and participants identification (*random effect*).

[a]SD; [b]variance, [c]R_c^2 (conditional coefficient of determination); [d]intraclass correlation coefficient

*$p<0.05$

HbA1c, glycated haemoglobin; LIPA: Light intensity physical activity; MVPA: Moderate-vigorous physical activity; SB: Sedentary behaviour; PHV: Peak Height Velocity; *B*: Unstandardized beta coefficient; *β:* Standardized beta coefficient; CI: Confident Interval; SD: standard deviation; SD^2: variance; R_c^2, conditional coefficient of determination; ICC, intraclass correlation coefficient.

ESM Table 8. *Results of multilevel compositional models examining associations between daily movement behaviours and interstitial glucose, adjusted for age and sex.*

Fixed effects	*B*	95% CI	β	*p* value
Z_{LIPA}				
ilr$_1$	0.919	-0.715, 2.582	0.063	0.285
ilr$_2$	-0.238	-1.114, 0.667	-0.043	0.603
ilr$_3$	-2.267	-4.923, 0.281	-0.150	0.091
Z_{MVPA}				
ilr$_1$	-0.531	-1.519, 0.479	-0.090	0.307
ilr$_2$	-2.357	-5.005, 0.182	-0.115	0.078
ilr$_3$	-0.452	-2.062, 1.135	-0.046	0.587
Z_{SB}				
ilr$_1$	1.657	-0.256, 3.634	0.217	0.101
ilr$_2$	0.022	-1.331, 1.424	0.003	0.974
ilr$_3$	1.815	-0.287, 4.001	0.123	0.101
Z_{sleep}				
ilr$_1$	-2.045	-4.591, 0.390	-0.147	0.110
ilr$_2$	0.251	-1.361, 1.853	0.018	0.763
ilr$_3$	-1.340	-2.572, -0.128	-0.239	0.036*
Sex	-0.095	-1.049, 0.858	-0.019	0.849
Age	-0.019	-0.211, 0.172	-0.021	0.844
Diabetes duration	-0.035	-0.170, 0.099	-0.050	0.616
Year of measurement	-0.028	-0.331, 0.270	-0.009	0.853
Daily insulin doses	3.395	2.101, 4.682	0.378	<0.001**
Carbohydrate rations	-0.066	-0.118, -0.015	-0.155	0.013*
Random effects				
Participants	1.757 [a]		3.086 [b]	
Model performance	0.741 [c]		0.679 [d]	

The Z_{LIPA} model is based on the set (*Z*) of coordinates (*ilr*) for LIPA: *ilr*$_1$ (LIPA at the expense of MVPA, SB and sleep), *ilr*$_2$ (MVPA at the expense of sleep and SB) and *ilr*$_3$ (sleep at the expense of SB)

The Z_{MVPA} model is based on the set (*Z*) of coordinates (*ilr*) for MVPA: *ilr*$_1$ (MVPA at the expense of LIPA, SB and sleep), *ilr*$_2$ (sleep at the expense of LIPA and SB) and *ilr*$_3$ (LIPA at the expense of SB)

The Z_{SB} model is based on the set (*Z*) of coordinates (*ilr*) for SB: *ilr*$_1$ (SB at the expense of LIPA, MVPA and sleep), *ilr*$_2$ (MVPA at the expense of LIPA and sleep) and *ilr*$_3$ (LIPA at the expense of sleep)

The Z_{Sleep} model is based on the set (*Z*) of coordinates (*ilr*) for sleep: *ilr*$_1$ (sleep at the expense of LIPA, MVPA and SB), *ilr*$_2$ (LIPA at the expense of MVPA and SB) and *ilr*$_3$ (MVPA at the expense of SB)

Models: adjusted for each set of coordinates (ilr), sex, age, disease duration, year of measurement, daily insulin doses, carbohydrate intake rations (fixed effects), and participants identification (random effect).

[a]SD; [b]variance, [c]R_c^2 (conditional coefficient of determination); [d]intraclass correlation coefficient

$^{*}p<0.05$, $^{**}p<0.001$

LIPA: Light intensity physical activity; MVPA: Moderate-vigorous physical activity; SB: Sedentary behaviour; PHV: Peak Height Velocity; *B*: Unstandardized beta coefficient; *β:* Standardized beta coefficient; CI: Confident Interval; SD: standard deviation; SD^2: variance; R_c^2, conditional coefficient of determination; ICC, intraclass correlation coefficient.

ESM Table 9. *Results of composition models for light physical activity, moderate-vigorous physical activity, sedentary behaviour, and sleep in relation to HbA_{1c} in each evaluation.*

	Baseline		*Follow-up 1*		*Follow-up 2*	
	B	*p* value	*B*	*p* value	*B*	*p* value
Z_{LIPA}						
ilr_1	2.433	0.730	22.281	0.044*	4.565	0.676
ilr_2	2.369	0.440	2.263	0.626	0.038	0.994
ilr_3	-6.680	0.514	-18.371	0.284	-44.141	0.004*
Z_{MVPA}						
ilr_1	1.422	0.700	-5.293	0.311	-1.485	0.817
ilr_2	-7.327	0.471	-26.791	0.121	-40.385	0.007*
ilr_3	-0.670	0.922	9.660	0.360	-18.332	0.087
Z_{SB}						
ilr_1	3.527	0.660	6.506	0.613	34.501	0.005*
ilr_2	2.756	0.589	-3.314	0.674	10.623	0.198
ilr_3	6.010	0.484	28.032	0.053	25.809	0.038*
Z_{sleep}						
ilr_1	-7.382	0.439	-23.494	0.152	-37.581	0.006*
ilr_2	-0.030	0.997	15.327	0.139	-8.445	0.436
ilr_3	-1.289	0.802	-7.225	0.356	-22.037	0.009*
PHV	-0.211	0.789	-0.318	0.812	-2.579	0.074
Diabetes duration	0.292	0.395	0.216	0.646	-0.035	0.946

The Z_{LIPA} model is based on the set (*Z*) of coordinates (*ilr*) for LIPA: ilr_1 (LIPA at the expense of MVPA, SB and sleep), ilr_2 (MVPA at the expense of sleep and SB) and ilr_3 (sleep at the expense of SB)

The Z_{MVPA} model is based on the set (*Z*) of coordinates (*ilr*) for MVPA: ilr_1 (MVPA at the expense of LIPA, SB and sleep), ilr_2 (sleep at the expense of LIPA and SB) and ilr_3 (LIPA at the expense of SB)

The Z_{SB} model is based on the set (*Z*) of coordinates (*ilr*) for SB: ilr_1 (SB at the expense of LIPA, MVPA and sleep), ilr_2 (MVPA at the expense of LIPA and sleep) and ilr_3 (LIPA at the expense of sleep)

The Z_{Sleep} model is based on the set (*Z*) of coordinates (*ilr*) for sleep: ilr_1 (sleep at the expense of LIPA, MVPA and SB), ilr_2 (LIPA at the expense of MVPA and SB) and ilr_3 (MVPA at the expense of SB)

Models: adjusted for each set of coordinates (ilr), PHV and disease duration.

* p-value < 0.05.

LIPA: Light intensity physical activity; MVPA: Moderate-vigorous physical activity; SB: Sedentary behaviour; PHV: Peak Height Velocity; *B*: Unstandardized beta coefficient

ESM Table 10. *Results of composition models for light physical activity, moderate-vigorous physical activity, sedentary behaviour, and sleep in relation to interstitial glucose in each evaluation.*

	Baseline		*Follow-up 1*		*Follow-up 2*	
	B	*p* value	*B*	*p* value	*B*	*p* value
Z_{LIPA}						
ilr_1	1.546	0.218	3.173	0.153	-0.645	0.777
ilr_2	0.260	0.662	1.356	0.156	-0.367	0.725
ilr_3	-4.348	0.025*	-8.779	0.021*	-6.252	0.047*
Z_{MVPA}						
ilr_1	-0.270	0.696	0.221	0.832	-0.131	0.916
ilr_2	-4.538	0.020*	-9.325	0.014*	-5.049	0.107
ilr_3	-0.836	0.480	-1.407	0.512	-3.759	0.085
Z_{SB}						
ilr_1	2.912	0.047*	5.471	0.049*	5.493	0.028*
ilr_2	0.743	0.428	2.168	0.192	1.803	0.268
ilr_3	3.512	0.031*	7.372	0.018*	2.494	0.355
Z_{sleep}						
ilr_1	-4.188	0.023*	-8.865	0.015*	-4.717	0.107
ilr_2	0.159	0.893	0.232	0.911	-2.352	0.283
ilr_3	-1.949	0.041*	-3.216	0.052	-3.444	0.038*
PHV	-0.115	0.430	-0.282	0.316	-0.322	0.310
Diabetes duration	0.030	0.626	-0.044	0.678	-0.105	0.378
Daily insulin doses	1.983	0.005*	4.371	0.003*	4.790	0.005*
Carbohydrate rations	-0.041	0.306	-0.210	0.001*	-0.128	0.037*

The Z_{LIPA} model is based on the set (*Z*) of coordinates (*ilr*) for LIPA: ilr_1 (LIPA at the expense of MVPA, SB and sleep), ilr_2 (MVPA at the expense of sleep and SB) and ilr_3 (sleep at the expense of SB)

The Z_{MVPA} model is based on the set (*Z*) of coordinates (*ilr*) for MVPA: ilr_1 (MVPA at the expense of LIPA, SB and sleep), ilr_2 (sleep at the expense of LIPA and SB) and ilr_3 (LIPA at the expense of SB)

The Z_{SB} model is based on the set (*Z*) of coordinates (*ilr*) for SB: ilr_1 (SB at the expense of LIPA, MVPA and sleep), ilr_2 (MVPA at the expense of LIPA and sleep) and ilr_3 (LIPA at the expense of sleep)

The Z_{Sleep} model is based on the set (*Z*) of coordinates (*ilr*) for sleep: ilr_1 (sleep at the expense of LIPA, MVPA and SB), ilr_2 (LIPA at the expense of MVPA and SB) and ilr_3 (MVPA at the expense of SB)

Models: adjusted for each set of coordinates (ilr), PHV, disease duration, daily insulin doses, and carbohydrate intake rations.

* p-value < 0.05.

LIPA: Light intensity physical activity; MVPA: Moderate-vigorous physical activity; SB: Sedentary behaviour; PHV: Peak Height Velocity; *B*: Unstandardized beta coefficient

ESM Table 11. *Results of linear regression models using ILR-transformed delta compositions of light physical activity, moderate-to-vigorous physical activity, sedentary behaviour, and sleep in relation to changes in HbA_{1c}.*

Fixed effects	*B*	95% CI	β	*p* value
Z_{LIPA}				
Δ ilr_1	-11.740	-35.570, 12.089	-0.148	0.326
Δ ilr_2	-7.968	-20.484, 4.547	-0.199	0.206
Δ ilr_3	-17.770	-45.039, 9.499	-0.196	0.196
Z_{MVPA}				
Δ ilr_1	-3.599	-19.712, 12.513	-0.080	0.655
Δ ilr_2	-8.526	-35.906, 18.852	-0.089	0.533
Δ ilr_3	-20.771	-42.192, 0.649	-0.355	0.057
Z_{SB}				
Δ ilr_1	22.179	0.840, 43.518	0.421	0.042
Δ ilr_2	4.023	-16.038, 24.085	0.075	0.688
Δ ilr_3	-3.001	-27.705, 21.702	-0.041	0.808
Z_{sleep}				
Δ ilr_1	-6.839	-32.813, 19.134	-0.092	0.598
Δ ilr_2	-14.870	-38.930, 9.188	-0.177	0.219
Δ ilr_3	-15.786	-30.437, -1.135	-0.380	0.035*
$PHV_{Baseline}$	0.401	-1.580, 2.383	0.059	0.685
Diabetes $duration_{Baseline}$	-0.361	-1.443, 0.721	-0.100	0.505

The Z_{LIPA} model is based on the set (*Z*) of coordinates (*ilr*) for LIPA: ilr_1 (LIPA at the expense of MVPA, SB and sleep), ilr_2 (MVPA at the expense of sleep and SB) and ilr_3 (sleep at the expense of SB)

The Z_{MVPA} model is based on the set (*Z*) of coordinates (*ilr*) for MVPA: ilr_1 (MVPA at the expense of LIPA, SB and sleep), ilr_2 (sleep at the expense of LIPA and SB) and ilr_3 (LIPA at the expense of SB)

The Z_{SB} model is based on the set (*Z*) of coordinates (*ilr*) for SB: ilr_1 (SB at the expense of LIPA, MVPA and sleep), ilr_2 (MVPA at the expense of LIPA and sleep) and ilr_3 (LIPA at the expense of sleep)

The Z_{Sleep} model is based on the set (*Z*) of coordinates (*ilr*) for sleep: ilr_1 (sleep at the expense of LIPA, MVPA and SB), ilr_2 (LIPA at the expense of MVPA and SB) and ilr_3 (MVPA at the expense of SB)

Models: adjusted for each set (*Z*) of coordinates (*ilr*), PHV, age, and disease duration.

* p-value < 0.05.

Δ, delta; HbA1c, glycated haemoglobin; LIPA: Light intensity physical activity; MVPA: Moderate-vigorous physical activity; SB: Sedentary behaviour; PHV: Peak Height Velocity; *B*: Unstandardized beta coefficient; *β:* Standardized beta coefficient; CI: Confident Interval.

ESM Table 12. *Results of linear regression models using ILR-transformed delta compositions of light physical activity, moderate-to-vigorous physical activity, sedentary behaviour, and sleep in relation to changes in interstitial glucose.*

Fixed effects	*B*	95% CI	β	*p* value
Z_{LIPA}				
Δ ilr_1	-2.329	-6.784, 2.125	-0.163	0.296
Δ ilr_2	-0.847	-2.811, 1.117	-0.136	0.388
Δ ilr_3	-3.440	-7.645, 0.763	-0.247	0.105
Z_{MVPA}				
Δ ilr_1	-0.022	-2.816, 2.771	-0.003	0.987
Δ ilr_2	-1.740	-6.094, 2.613	-0.116	0.423
Δ ilr_3	-3.866	-7.690, -0.042	-0.404	0.047*
Z_{SB}				
Δ ilr_1	3.985	0.626, 7.343	0.483	0.021*
Δ ilr_2	1.385	-2.084, 4.855	0.165	0.424
Δ ilr_3	-0.425	-4.676, 3.824	-0.035	0.840
Z_{sleep}				
Δ ilr_1	-1.633	-5.664, 2.397	-0.144	0.417
Δ ilr_2	-3.048	-7.500, 1.404	-0.200	0.174
Δ ilr_3	-2.453	-4.758, -0.149	-0.380	0.037*
$PHV_{Baseline}$	0.014	-0.320, 0.348	0.013	0.932
Diabetes $duration_{Baseline}$	-0.111	-0.294, 0.070	-0.192	0.221
Daily insulin $doses_{Baseline}$	-1.005	-2.881, 0.870	-0.151	0.284
Carbohydrate $rations_{Baseline}$	-0.093	-0.225, 0.038	-0.216	0.161

The Z_{LIPA} model is based on the set (*Z*) of coordinates (*ilr*) for LIPA: ilr_1 (LIPA at the expense of MVPA, SB and sleep), ilr_2 (MVPA at the expense of sleep and SB) and ilr_3 (sleep at the expense of SB)

The Z_{MVPA} model is based on the set (*Z*) of coordinates (*ilr*) for MVPA: ilr_1 (MVPA at the expense of LIPA, SB and sleep), ilr_2 (sleep at the expense of LIPA and SB) and ilr_3 (LIPA at the expense of SB)

The Z_{SB} model is based on the set (*Z*) of coordinates (*ilr*) for SB: ilr_1 (SB at the expense of LIPA, MVPA and sleep), ilr_2 (MVPA at the expense of LIPA and sleep) and ilr_3 (LIPA at the expense of sleep)

The Z_{Sleep} model is based on the set (*Z*) of coordinates (*ilr*) for sleep: ilr_1 (sleep at the expense of LIPA, MVPA and SB), ilr_2 (LIPA at the expense of MVPA and SB) and ilr_3 (MVPA at the expense of SB)

Models: adjusted for each set of coordinates (ilr), PHV, disease duration, daily insulin doses, and carbohydrate intake rations.

* p-value < 0.05.

Δ, delta; LIPA: Light intensity physical activity; MVPA: Moderate-vigorous physical activity; SB: Sedentary behaviour; PHV: Peak Height Velocity; *B*: Unstandardized beta coefficient; *β:* Standardized beta coefficient; CI: Confident Interval.

ESM Table 13. *Results of multilevel composition models for light physical activity, moderate-vigorous physical activity, sedentary behaviour, and sleep in relation to HbA_{1c} in those subjects who completed the entire study (n=62).*

Fixed effects	*B*	95% CI	β	*p* value
Z_{LIPA}				
ilr_1	-0.303	-10.095, 9.849	-0.004	0.952
ilr_2	-1.314	-6.106, 3.609	-0.046	0.596
ilr_3	-17.502	-31.829, -3.600	-0.242	0.016*
Z_{MVPA}				
ilr_1	-1.138	-6.795, 4.547	-0.037	0.698
ilr_2	-14.795	-29.394, -0.770	-0.158	0.042*
ilr_3	-9.378	-18.543, -0.072	-0.186	0.049*
Z_{SB}				
ilr_1	15.011	4.415, 25.753	0.383	0.007*
ilr_2	4.100	-3.466, 11.737	0.114	0.297
ilr_3	8.123	-3.865, 20.685	0.108	0.190
Z_{sleep}				
ilr_1	-13.569	-27.416, -0.267	-0.199	0.048*
ilr_2	-5.119	-14.548, 4.491	-0.072	0.297
ilr_3	-9.889	-16.803, -3.039	-0.341	0.006*
PHV	-1.159	-2.711, 0.391	-0.179	0.152
Diabetes duration	0.116	-0.625, 0.858	0.035	0.762
Year of measurement	1.638	-0.112, 3.375	0.110	0.070
Random effects				
Participants	9.409 [a]		88.530 [b]	
Model performance	0.623 [c]		0.601 [d]	

The Z_{LIPA} model is based on the set (*Z*) of coordinates (*ilr*) for LIPA: ilr_1 (LIPA at the expense of MVPA, SB and sleep), ilr_2 (MVPA at the expense of sleep and SB) and ilr_3 (sleep at the expense of SB)

The Z_{MVPA} model is based on the set (*Z*) of coordinates (*ilr*) for MVPA: ilr_1 (MVPA at the expense of LIPA, SB and sleep), ilr_2 (sleep at the expense of LIPA and SB) and ilr_3 (LIPA at the expense of SB)

The Z_{SB} model is based on the set (*Z*) of coordinates (*ilr*) for SB: ilr_1 (SB at the expense of LIPA, MVPA and sleep), ilr_2 (MVPA at the expense of LIPA and sleep) and ilr_3 (LIPA at the expense of sleep)

The Z_{Sleep} model is based on the set (*Z*) of coordinates (*ilr*) for sleep: ilr_1 (sleep at the expense of LIPA, MVPA and SB), ilr_2 (LIPA at the expense of MVPA and SB) and ilr_3 (MVPA at the expense of SB)

Models: adjusted for each set (*Z*) of coordinates (*ilr*), PHV, disease duration, year of measurement (*fixed effects*) and participants identification (*random effect*).

* p-value < 0.05.

HbA1c, glycated haemoglobin; LIPA: Light intensity physical activity; MVPA: Moderate-vigorous physical activity; SB: Sedentary behaviour; PHV: Peak Height Velocity; *B*: Unstandardized beta coefficient; *β:* Standardized beta coefficient; CI: Confident Interval; SD: standard deviation; SD^2: variance; R_c^2, conditional coefficient of determination; ICC, intraclass correlation coefficient.

ESM Table 14. *Results of multilevel composition models for light physical activity, moderate-vigorous physical activity, sedentary behaviour, and sleep in relation to interstitial glucose in those subjects who completed the entire study (n=62).*

Fixed effects	*B*	95% CI	β	*p* value
Z_{LIPA}				
ilr_1	1.063	-0.674, 2.839	0.070	0.244
ilr_2	-0.272	-1.204, 0.685	-0.047	0.573
ilr_3	-2.857	-5.677, -0.177	-0.180	0.040*
Z_{MVPA}				
ilr_1	-0.611	-1.628, 0.420	-0.100	0.252
ilr_2	-2.930	-5.801, -0.206	-0.134	0.038*
ilr_3	-0.638	-2.287, 0.979	-0.062	0.452
Z_{SB}				
ilr_1	2.106	0.131, 4.169	0.263	0.042*
ilr_2	0.096	-1.282, 1.516	0.013	0.893
ilr_3	2.218	-0.071, 4.622	0.139	0.063
Z_{sleep}				
ilr_1	-2.558	-5.309, 0.049	-0.174	0.058
ilr_2	0.223	-1.428, 1.864	0.015	0.794
ilr_3	-1.664	-2.969, -0.398	-0.285	0.013*
PHV	-0.109	-0.403, 0.183	-0.080	0.475
Diabetes duration	-0.040	-0.188, 0.106	-0.056	0.598
Year of measurement	-0.018	-0.352, 0.311	-0.005	0.914
Daily insulin doses	3.423	2.019, 4.824	0.374	<0.001**
Carbohydrate rations	-0.066	-0.123, -0.011	-0.149	0.021*
Random effects				
Participants	1.856 [a]		3.445 [b]	
Model performance	0.753 [c]		0.691 [d]	

The Z_{LIPA} model is based on the set (*Z*) of coordinates (*ilr*) for LIPA: ilr_1 (LIPA at the expense of MVPA, SB and sleep), ilr_2 (MVPA at the expense of sleep and SB) and ilr_3 (sleep at the expense of SB)

The Z_{MVPA} model is based on the set (*Z*) of coordinates (*ilr*) for MVPA: ilr_1 (MVPA at the expense of LIPA, SB and sleep), ilr_2 (sleep at the expense of LIPA and SB) and ilr_3 (LIPA at the expense of SB)

The Z_{SB} model is based on the set (*Z*) of coordinates (*ilr*) for SB: ilr_1 (SB at the expense of LIPA, MVPA and sleep), ilr_2 (MVPA at the expense of LIPA and sleep) and ilr_3 (LIPA at the expense of sleep)

The Z_{Sleep} model is based on the set (*Z*) of coordinates (*ilr*) for sleep: ilr_1 (sleep at the expense of LIPA, MVPA and SB), ilr_2 (LIPA at the expense of MVPA and SB) and ilr_3 (MVPA at the expense of SB)

Models: adjusted for each set of coordinates (ilr), PHV, disease duration, year of measurement, daily insulin doses, carbohydrate intake rations (fixed effects), and participants identification (random effect).

* *p*-value < 0.05, ** *p*-value < 0.001.

LIPA: Light intensity physical activity; MVPA: Moderate-vigorous physical activity; SB: Sedentary behaviour; PHV: Peak Height Velocity; *B*: Unstandardized beta coefficient; *β:* Standardized beta coefficient; CI: Confident Interval; SD: standard deviation; SD^2: variance; R_c^2, conditional coefficient of determination; ICC, intraclass correlation coefficient

ESM Fig. 1. *Average time of the three evaluated moments dedicated to each movement pattern. LIPA: Light intensity physical activity; MVPA: Moderate-vigorous physical activity; SB: Sedentary behaviour.*

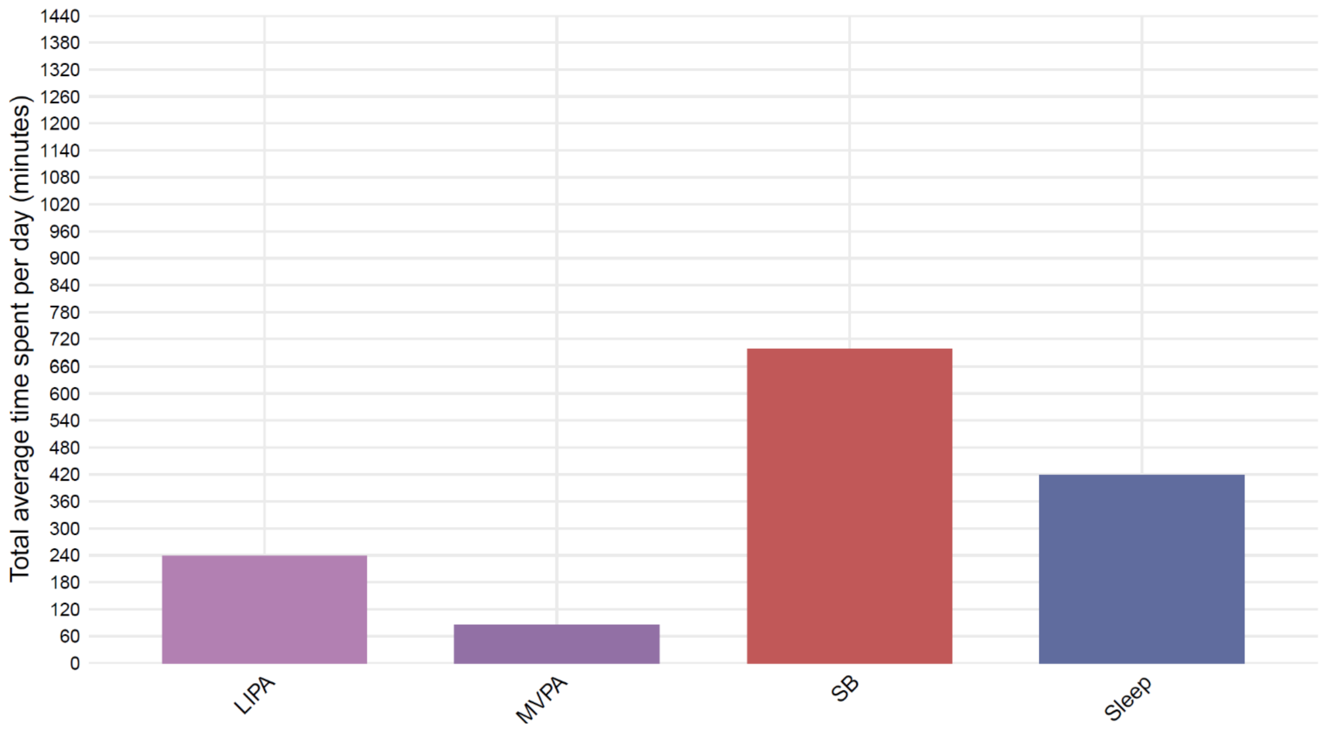

Supplement: Supplementary file 1 — ESM (PDF 615 KB) [file 125_2025_6496_MOESM1_ESM.pdf]
